# Using Virtual Reality to Assess Ethical Decisions in Road Traffic Scenarios: Applicability of Value-of-Life-Based Models and Influences of Time Pressure

**DOI:** 10.3389/fnbeh.2017.00122

**Published:** 2017-07-05

**Authors:** Leon R. Sütfeld, Richard Gast, Peter König, Gordon Pipa

**Affiliations:** Neuroinformatics, Institute of Cognitive Science, Osnabrück UniversityOsnabrück, Germany

**Keywords:** self-driving cars, moral judgment, ethical decisions, modeling, virtual reality, value-of-life scale, time pressure

## Abstract

Self-driving cars are posing a new challenge to our ethics. By using algorithms to make decisions in situations where harming humans is possible, probable, or even unavoidable, a self-driving car's ethical behavior comes pre-defined. *Ad hoc* decisions are made in milliseconds, but can be based on extensive research and debates. The same algorithms are also likely to be used in millions of cars at a time, increasing the impact of any inherent biases, and increasing the importance of getting it right. Previous research has shown that moral judgment and behavior are highly context-dependent, and comprehensive and nuanced models of the underlying cognitive processes are out of reach to date. Models of ethics for self-driving cars should thus aim to match human decisions made in the same context. We employed immersive virtual reality to assess ethical behavior in simulated road traffic scenarios, and used the collected data to train and evaluate a range of decision models. In the study, participants controlled a virtual car and had to choose which of two given obstacles they would sacrifice in order to spare the other. We randomly sampled obstacles from a variety of inanimate objects, animals and humans. Our model comparison shows that simple models based on one-dimensional value-of-life scales are suited to describe human ethical behavior in these situations. Furthermore, we examined the influence of severe time pressure on the decision-making process. We found that it decreases consistency in the decision patterns, thus providing an argument for algorithmic decision-making in road traffic. This study demonstrates the suitability of virtual reality for the assessment of ethical behavior in humans, delivering consistent results across subjects, while closely matching the experimental settings to the real world scenarios in question.

## Introduction

Privately owned cars with autopilots first became a reality with a software update which Tesla Motors released to its fleet in October 2015, and many comparable systems will be on the market soon. While initially, these systems are likely to be restricted to highway use, they will eventually make their way into cities, with estimates predicting autonomous vehicles (AVs) dominating road traffic by the 2040s (Marcus, 2012; Litman, 2014). The new technology is expected to reduce the number of car accidents significantly: The German Federal Statistics Agency reports that in 2015, 67% of all accidents with injuries to people were caused by driver misconduct. A 2008 survey by the National Highway Traffic Safety Administration (NHTSA) even showed that human error played a crucial role in 93% of traffic accidents in the US. These numbers outline the enormous potential of self-driving cars regarding road safety. In fact, Johansson and Nilsson (2016) claim that self-driving cars will adjust their driving style and speed such that safe handling of any unexpected event is guaranteed at all times. However, this approach appears unrealistic for many mixed traffic (human and AVs) and inner city scenarios. To ensure absolute safety even in very unlikely events, the car would have to drive in an overly cautious manner, and as a result may be switched off by many drivers, or tempt other drivers to engage in risky overtaking. Other rare events, such as a distracted human driver swerving into the opposite lane, seem very hard to evade altogether under any circumstances. Even when completely taking human drivers out of the equation, we are left with a considerable number of accidents, caused, for instance, by technical or engineering failure (Goodall, 2014b). Altogether, with over a billion cars in operation worldwide, the sheer amount of traffic virtually guarantees that, in spite of the overall expected reduction of accidents, critical situations will occur on a daily basis.

With accidents involving autonomous cars being and becoming a reality, ethical considerations will inevitably come into play. Any decision that involves risk of harm to a human or even an animal is considered to be an ethical decision. This includes everyday decisions, e.g., deciding if and when to take a minor risk in overtaking a cyclist. But it also includes quite rare situations in which a collision is unavoidable, but a decision can be made as to which obstacle to collide with. By relying on algorithms to make these decisions, a self-driving car's ethics come pre-defined by the engineer, whether it's done with sophisticated ethical systems or simple rules such as “always stay in the lane.” This development poses a new challenge to the way we handle ethics. If human drivers are in an accident and make a bad decision from an ethical standpoint, we count in their favor that they had incomplete knowledge of the situation and only fractions of a second to make a decision. Therefore, we typically refrain from assigning any blame to them, morally or legally (Gogoll and Müller, 2017). Algorithms in self-driving cars, on the other hand, can estimate the potential outcome of various options within milliseconds, and make a decision that factors in an extensive body of research, debates, and legislations (Lin, 2013). Moreover, the same algorithms are likely to be used in thousands or millions of cars at a time. Situations that are highly unlikely for an individual car become highly probable over the whole fleet. This enhances the importance of getting it right, and unpreparedness to handle this type of situation may result in a significant number of bad decisions overall.

Ultimately, moral decision-making systems should be considered a necessity for self-driving cars (Goodall, 2014a). The present study addresses the question of how to assess and how to model human moral decision-making in situations in which a collision is unavoidable and a decision has to be made as to which obstacle to collide with. We conducted a virtual reality (VR) study in which participants had to make exactly this type of decision for varying combinations of obstacles, and used the obtained data to train and evaluate a number of different ethical decision-making models. In the next section, we will review the current state of psychological research with respect to moral judgment and decision-making, and derive the outline for the present experiment.

### The psychology of moral judgment

The scenario in this study can be seen as an adaptation of the trolley dilemma, a thought experiment commonly used in research on moral judgment and decision-making, in which a runaway trolley is heading toward a group of five people. The only way to save these five is to pull a lever and divert the trolley onto a different track, killing a single person instead (Thomson, 1985). The utilitarian choice here is to pull the lever and sacrifice one person in order to save five. By contrast, deontologism focuses on the rights of individuals, often putting these ahead of utilitarian considerations. From this perspective, the act of killing a person would be considered morally wrong, even if it means saving five other lives. In a popular alteration of the trolley problem, called the footbridge dilemma, the subject finds themself on a bridge over the tracks with a stranger. Pushing the stranger off the bridge in front of the oncoming train would stop the train and save the five people standing on the track. Interestingly, most people say they would pull the lever in the original trolley dilemma, but only a minority also says they would push the stranger off the bridge in the footbridge dilemma (Greene et al., 2001). An extensive body of psychological research is concerned with the affective, cognitive and social mechanisms underlying this judgment, our ethical intuitions and behavior (Huebner et al., 2009; Christensen and Gomila, 2012; Cushman and Greene, 2012; Waldmann et al., 2012; Avramova and Inbar, 2013). Most prominently, the dual process theory, put forward by Greene et al. (2004), proposes two distinct cognitive systems in competition. The first is an intuitive, emotionally rooted system, eliciting negative affect when behavioral rules are violated, favoring decisions in line with deontological ethics. The second one is a controlled, reasoning-based system, favoring decisions corresponding with utilitarian ethics. Greene's dual-process theory thus explains the different endorsement rates of utilitarian behavior in the trolley and footbridge dilemma by the more emotionally engaging nature of the latter. Pushing a stranger off a bridge instead of pulling a lever requires personal force and uses harm as a means to an end, rather than as a side effect, both increasing the emotionality of the situation, and thus shifting focus to the system favoring deontological ethics (Greene et al., 2001). Similarly, framing a dilemma as more personal (“I would do…” instead of “it is acceptable to…”) and increasing the emotional proximity to the potential victim will also result in fewer utilitarian choices (Greene et al., 2001; Tassy et al., 2013). Neuroscientific evidence is provided by Tassy et al. (2012), showing that disrupting the right dorsolateral prefrontal cortex, associated with emotional processing, increases the likelihood of utilitarian responses. Valdesolo and DeSteno (2006) found an increased probability of utilitarian responses when inducing positive affect, arguing that the positive affect may cancel out the negative affect connected to rule violations.

However, the dual-process theory, based on the emotion-cognition distinction is not undisputed. Cushman (2013) argues that while a distinction between competing processes is necessary, the distinction between affective and non-affective processing is inadequate, since both processes must involve cognition, as well as affective content. Instead, he proposes a distinction based on two cognitive mechanisms borrowed from the field of reinforcement learning. The first is an action-based system, assigning reward values to possible actions in a given situation. These reward values are learned from experience and statically assigned to a given situation-action-pair. The second mechanism is outcome-based and relies on an underlying world model. In simplified terms, it predicts the consequences of the possible actions in a given situation and reassigns the value of the consequence to the action that leads to it. In the trolley dilemma, the outcome-based system would favor utilitarian behavior, and the action-based system would not intervene because the action of pulling a lever is not generally associated with negative reward. Conversely, the action of pushing a person off a bridge is associated with negative reward, thus explaining the lower endorsement rates of utilitarian behavior in the footbridge dilemma. Further evidence in favor of the action vs. outcome distinction in dual-process models is given, e.g., in Cushman et al. (2012) and Francis et al. (2016).

In another theory in the realm of moral judgment, Haidt and Graham (2007) aim to explain different views of opposing political camps (liberals and conservatives) with a model of morality based on five factors, and the relative importance of each of these factors to members of the political camps. Finally, based on a large body of neuroscientific evidence, Moll et al. (2008) propose a detailed account of moral emotions as the foundation of our moral judgment. While none of the two entail concrete predictions with respect to moral decision-making in the trolley dilemma and similar scenarios, they demonstrate that the scope of the dual-process theories is limited, and that we are a long way from a comprehensive theory about the cognitive mechanisms governing our moral judgment and behavior.

### Virtual reality assessment and effects of time contraints

While most of the aforementioned research relies on abstract, text-based presentations of dilemma situations, a growing number of studies makes use of the possibilities provided by virtual reality (VR) technology. VR, and in particular immersive VR using head-mounted displays (HMDs) and head-tracking, allows assessing moral behavior in a naturalistic way, immersing the subject in the situation, providing much richer contextual information, and allowing for more physical input methods. In an immersive VR version of the trolley dilemma, Navarrete et al. (2012) were able to confirm the utilitarian choice's approval rate of 90%, previously found in text-based studies. Further, they found a negative correlation between emotional arousal and utilitarian choices, in line with the predictions of the dual process theory. In contrast to this, Patil et al. (2014) found both emotional arousal and endorsements of utilitarian choices to be higher in a desktop-VR setting with 3D graphics on a desktop screen than in a text-based setting. While hinting toward a possible distinction between moral judgment and behavior, the results also suggest that features other than emotional arousal play a major role in our moral judgment. The authors argue that the contextual saliency (including a depiction of the train running over the virtual humans) may have shifted the subjects' focus from the action itself toward the outcome of their decision. The tendency to favor utilitarian judgment would then fit Cushman's account of the dual-process theory. In a similar study by Francis et al. (2016), participants were confronted with the footbridge dilemma, either in an immersive VR environment or in a text-based scenario. In the text-based condition, endorsement of the utilitarian choice was low at around 10%, in line with expectations based on previous assessments. In the VR condition, however, subjects opted to push the stranger off the bridge in up to 70% of the trials. These results are again in line with Cushman's account of the dual-process theory, and make a strong case for the notion of moral judgment and moral behavior being distinct constructs. In a different approach, Skulmowski et al. (2014) varied the standard design of the trolley dilemma in multiple ways. First, they virtually placed participants in the trolley's cockpit instead of a bystanders' perspective. Second, they designed the track to split into three and blocked the middle track with a stationary trolley, which had to be avoided. Participants were thus forced to choose between the outside tracks, precluding the deontological option of not intervening in the situation. Third, the subjects had to react within 2.5 s after the obstacles became visible. Finally, in addition to varying the number of people on the available tracks, the authors added a number of trials with only one person standing on either of the available tracks. These differed in gender, ethnicity, and whether the person was facing toward the trolley or away from it. Unsurprisingly, the group was saved in 96% of the the one-vs.-many trials. In the single obstacle trials, significant differences were only found in the gender condition (deciding between a man and a woman), with men being sacrificed in around 58% of the cases.

The natural passing of time is a feature inherent to VR studies of this kind. While in principle, it would be possible to pause time in the virtual world, doing so might break immersion and would likely lessen the ecological validity of the obtained results. The previously mentioned studies all imposed some time constraints, but no systematic variation of response time windows was performed. Nevertheless, the dual-process theories would predict time pressure to influence our moral judgment. The action-based system in Cushman's account of the dual-process theory is thought to be simple and quick, while the outcome-based system involves higher cognitive load and is ultimately slower. Greene's account of the dual-process theory suggests that in emotionally engaging dilemmas, the controlled cognitive system needs to override the initial emotional response before making a utilitarian judgment (Greene, 2009). Indeed, increased cognitive load during decision time was shown to increase response times in personal dilemmas, when a utilitarian response was given (Greene et al., 2008). Paxton et al. (2012) showed that moral judgments can be changed with persuasive arguments, but additional time for deliberation was required for the change to occur. To the best of our knowledge, so far only one study systematically varied the length of response time windows. In Suter and Hertwig (2011), participants were either restricted to give a response within 8 s, or they had to first deliberate for 3 min. For high-conflict scenarios, such as the footbridge dilemma, higher time pressure led to fewer utilitarian responses. A second experiment in the same study supports this finding. When no time limitations were given, but one group was instructed to respond intuitively, and the other group was instructed to deliberate before entering a reaction, the intuitive group's response times were a lot shorter than the deliberate group's, and they gave fewer utilitarian responses.

In conclusion, VR studies have shown the importance of contextual cues for our decision-making and provide intriguing evidence for a distinction of moral judgment and behavior. Moreover, time constraints, as an inherent feature to VR setups, have been recognized as a factor in our moral decision-making. There is evidence suggesting that longer deliberation may facilitate utilitarian decisions in certain complex scenarios, but we still lack a systematic analysis of the influence of time pressure on moral judgment.

### Modeling of human moral behavior

An important criterion that an ethical decision-making system for self-driving cars or other applications of machine ethics should meet is to make decisions in line with those made by humans. While complex and nuanced ethical models capable of replicating our cognitive processes are out of reach to date, simpler models may deliver adequate approximations of human moral behavior, when the scope of the model is confined to a small and specific set of scenarios. Value-of-life-based models stand to reason as a possible solution for any situation in which a decision has to be made as to which one of two or more people, animals, other obstacles, or groups thereof to collide with. An account of a value-of-life model that is focused on a person's age is given by Johansson-Stenman and Martinsson (2008). The authors conducted a large-scale survey in which people had to indicate in several instances, on which of two road-safety-improvement measures they would rather spend a given budget. The available measures differed with respect to the age and expected number of people that would be saved, as well as whether the ones saved would be pedestrians or car drivers. The authors used a standard probit regression model to fit the observed data, and found that not the number of saved lives, but rather the number of saved life-years to be the most important factor in the subjects' decision, allowing for specific values of life to be assigned to each age group. Beyond this, they found pedestrians to be valued higher than car drivers of the same age, indicating consent as a factor in the valuation. While discriminating between potential human crash victims based on age, or possibly gender, is unlikely to gain general public acceptance, Goodall (2014a) suggests using value-of-life scales in cases where higher-level rules fail to provide the system with clear instructions. Furthermore, if we take animals into account, value-of-life scales stand to reason as a way of dealing with vastly differing probabilities. When a decision has to be made between killing a dog with near certainty and taking a 5% risk of injuring a human, how should the algorithm decide? We don't seem to take much issue with assigning different values of life to different species, and a system favoring pets over game or birds might be acceptable in the public eye. While this makes the case for at least some form of value-of-life model, it remains to be seen to what extent such models are able to capture the complexity of human ethical decision-making.

### Deriving and outlining the experiment

As discussed in previous sections, our moral judgment is highly dependent on a wide variety of contextual factors, and there is no ground truth in our ethical intuitions that holds irrespective of context. We thus argue that any implementation of an ethical decision-making system for a specific context should be based on human decisions made in the same context. To date, our limited understanding of the cognitive processes involved prevents us from constructing a comprehensive ethical model for use in critical real-world scenarios. In the context of self-driving cars, value-of-life scales stand to reason as simple models of human ethical behavior when a collision is unavoidable, and an evaluation of their applicability in this context is the main focus of this study.

To this end, participants were placed in the driver's seat of a virtual car heading down a road in a suburban setting. Immersive VR technology was used to achieve a maximum in perceived presence in the virtual world. A wide variety of different obstacles, both animate and inanimate, were randomly paired and presented in the two lanes ahead of the driver. Participants had to decide which of the two they would save, and which they would run over. Since prolonged sessions in immersive VR can cause nausea and discomfort, we opted for a pooled experimental design with short sessions of 9 trials per condition and participant. We thus pooled the trials of all participants, and used this data set to train three different logistic regression models to predict the lane choice for a given combination of obstacles. (1) The pairing model uses each possible pairing of obstacles as a predictor. Here, a given prediction reflects the frequency with which one obstacle was chosen over the other in the direct comparisons. Since an obstacle is not represented with a single numerical value, the pairing model is not a value-of-life model, but serves as a frame of reference. (2) The obstacle model assigns one coefficient to each obstacle and uses the obstacles' occurrences as predictors. We interpret these coefficients as the obstacle's value of life. (3) The cluster model uses only one coefficient per category of obstacles, as they resulted from a clustering based on the frequency with which each obstacle was hit.

We compare the three different models to test a set of hypotheses. Our first hypothesis was that a one-dimensional value-of-life-based model (i.e., the obstacle model) fully captures the complexities of pairwise comparisons. The obstacle model should thus be as accurate as the pairing model. This would mean that our ethical decisions can be described by a simple model in which each possible obstacle is represented by a single value, and the decision which obstacle to save is based only on these respective values. We further hypothesized (Hypothesis 2) that within-category distinctions, for example, between humans of different age, are an important factor in the decisions. Specifically, the obstacle model should prove to be superior to the cluster model. Furthermore, since a certain amount of time pressure is inherent to naturalistic representations of this scenario, we investigated its influence on the decisions by varying the time to respond in two steps, giving participants a response window of either 1 or 4 s. We found 4 s of decision time to induce relatively little time pressure in the used scenario, while one second still left a sufficient amount of time to comprehend and react. We hypothesized (Hypothesis 3) that more errors would be made under increased time pressure, and that ethical decisions would thus be less consistent across subjects in these trials. The dual-process theories would predict a higher endorsement of utilitarian choices with more time to deliberate (i.e., in the slow condition). However, for comparisons of single obstacles, there is no clearly defined utilitarian choice. If anything, basing the decision in human vs. human trials on a person's expected years to live could be considered utilitarian, and is partly covered in Hypothesis 2. Moreover, the omission of a lane change, despite running over the more valuable obstacle, could be interpreted as a deontological choice, but we didn't formulate any directional hypothesis regarding this factor prior to the study.

## Methods

**Figure 1:**
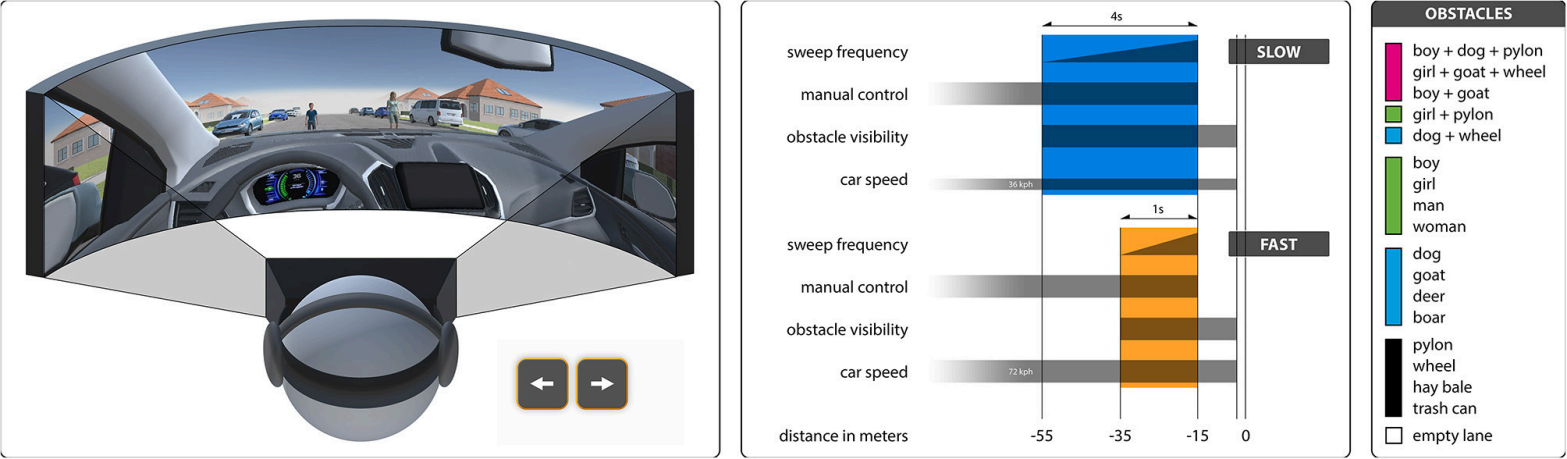
**(Left)** Overview of the experimental setting. **(Middle)** Timelines of the slow and fast conditions. **(Right)** Overview of all obstacles used. Colors indicate cluster assignments.

The experiment ran in a 3D virtual reality application, implemented with the Unity game engine, using the Oculus Rift DK2 as the head-mounted display. The audio was played through Bose QC25 and Sennheiser HD215 headphones throughout the experiment. The participants were sitting in the driver's seat of a virtual car heading down a suburban road. Eventually, two obstacles, one on either lane, blocked the car's path and the participants had to choose which of the two obstacles to hit. Using the arrow keys on the keyboard, the participants were able to switch between the two lanes at all times, up to a point approximately 15 m before impact. This way, we provided a high level of agency, intended to closely resemble manual car driving, while making sure the decision could not be avoided by zig-zagging in the middle of the road or crashing the car before reaching the obstacles. We used 17 different obstacles from three different categories, i.e., inanimate objects, animals, and humans as single obstacles, as well as composite obstacles. An empty lane was additionally used as a control. For each trial, two of the 17+1 obstacles were pseudo-randomly chosen and allocated to the two lanes, as was the starting lane of the participant's car. A wall of fog at a fixed distance from the participant's point of view controlled the exact time of the obstacle's onset. We varied the length of the reaction time window by varying the fog distance and car speed, resulting in a window of 4 s for the slow, and one second for the fast condition. To indicate how much time was left to make a decision at each point in time, a low-to-high sweep sound was played as an acoustic cue. The sound started and ended on the same respective frequencies in both conditions, thus sweeping through the frequency band quicker in the fast condition. After the decision time window had ended around 15 meters before impact, the car kept moving, completing any last instant lane changes. Right before impact, all movement was frozen, all sounds stopped, and the screen faded to black, marking the end of a trial. Figure 1 illustrates the chronological progression of the trials in the fast and slow condition, and gives an overview of all obstacles. The fast and slow trials were presented in separate blocks of 9 trials each. Two more blocks of trials were presented but not analyzed in the current study, and all four blocks were presented in randomized order. We chose obstacle pairings such that each obstacle typically appeared once per subject and block. The frequency of appearance of all 153 possible pairings, as well as the lane allocations and starting lanes, were balanced over all subjects.

### Sample and timeline

Our sample consists of 105 participants (76 male, 29 female) between the age of 18 and 60 (mean: 31) years. We excluded one subject who reported a partial misunderstanding of the task, as well as three outliers whose decisions were the opposite of the model prediction (see below) in more than 50% of their respective trials. Most of the participants were university students or visitors of the GAP9 philosophy conference. Before participating, we informed all subjects about the study, potential risks and the option to abort the experiment at any time. They were also informed that the external screens would be turned off during the experiment, so that others could not observe their decisions. After signing a consent form, they were asked to put on the HMD and headphones, and then received all further information within the application. As a first task, they had to complete a training trial, avoiding three pylons by alternating between the lanes. Upon hitting a pylon, the training trial was repeated until completed without error. This procedure gave participants a chance to familiarize themselves with the VR environment, and it made sure they had understood the controls before entering the experimental trials. The study conformed to the Code of Ethics of the American Psychological Association, as well as to national guidelines, and was approved by the Osnabrück University's ethics committee.

## Results

We pooled all data and did not consider between-subject differences in the analysis. In the experimental trials, the mean number of lane switches per trial was 0.816 in the slow and 1.037 in the fast condition. We estimated error rates for both conditions, using trials in which one of the lanes was empty. Hitting the only obstacle in such a trial was considered an error, as we find it safe to assume that the outcome in these trials is a result of inadvertently pressing the wrong button, instead of a meaningful decision. This event occurred in 2.8% of all trials containing an empty lane in the slow condition, and in 12.0% of the relevant trials in the fast condition. As a frame of reference, the chance level for this was at 50%.

### Behavioral models

All models used in the present study were logistic regression models, using the occurrence of obstacle pairings, individual obstacles or clusters, i.e., obstacle categories (see below) in a particular trial as predictors for the lane choice. Furthermore, we added a constant offset and the trial's starting lane as predictors to all models. The constant offset allowed us to detect potential biases in the overall lane preference (left or right). Such a bias could occur, for example, when participants are used to right-hand traffic and feel that using the right-hand lane is generally more acceptable. Including the starting lane as a predictor allowed us to detect a bias to stay in the respective trials' starting lane—we would label this an omission bias—or to move away from the starting lane, i.e., a panic reaction bias.

A model's predicted probability of choosing to drive in the left lane is given by $p\left(Y=\text{left}|X\right)=\frac{1}{1+\exp\left(-X\right)}$, with *X* being the model-specific representation of a particular trial.

**Figure 2:**
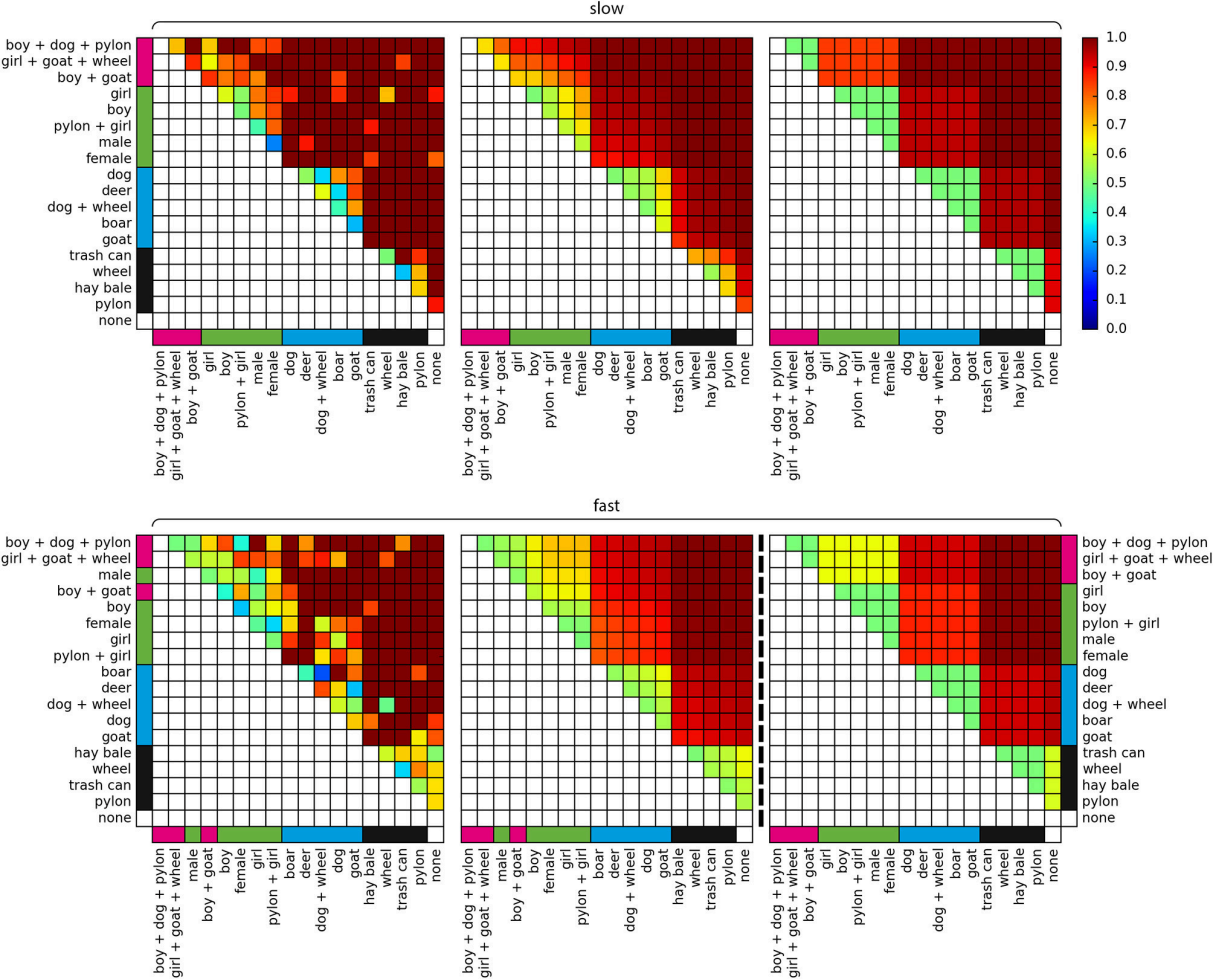
Model predictions. **(Top)** Slow condition, **(Bottom)** fast condition, **(Left)** pairing model, **(Middle)** obstacle model, **(Right)** cluster model. Colors indicate the probability of saving the row-obstacle (y-axis) and sacrificing the column-obstacle (x-axis). Pink, green, blue, and black bars indicate cluster assignments based on agglomerative clustering in the slow condition (see Figure 3). For means of comparability, the cluster model in the fast condition was fit with the semantic cluster assignments from the slow condition.

In the pairing model, a trial is represented as *X*_*p*_ = *cω*_*i*_ + *sω*_*s*_ + ω_*b*_, where ω_*i*_ is the coefficient for obstacle pairing *i* (e.g., {boy, woman}), *c* ∈ {−1, 1} is the lane configuration in the respective trial (e.g., 1 if the boy is in the left lane, −1 if the woman is in the left lane), ω_*s*_ is the starting lane coefficient, *s* ∈ {−1, 1} is the starting lane −1 if the starting lane is right, and ω_*b*_ is the coefficient for the lane bias. The model is thus making a prediction based on a general preference for one of the lanes, the starting lane of the respective trial, and which of the 153 possible pairings is presented in the trial, resulting in 155 parameters in total. Figure 2 left shows the predictions of the pairing model. Since each pairing of obstacles has its own free parameter, the model allows for intransitive and other complex relations between the obstacles. For example, in the slow condition, the pairing model deems the goat to be more valuable than the boar, and the boar to be more valuable than the deer, but the goat to be *less* valuable than the deer. Consequentially, there is no single value of life for an obstacle in the pairing model. An obstacle's value is only defined relative to each of the other obstacles.

In the obstacle model, a trial is represented as *X*_*o*_ = ω_*ro*_ − ω_*lo*_ + *sω*_*s*_ + ω_*b*_, with ω_*ro*_ and ω_*lo*_ being the coefficients for the right and left obstacle in the respective trial. Each obstacle is thus represented by a single characteristic value or value of life. All pairwise comparisons result directly from a subtraction of the respective two values of life. Thus, when sorting all obstacles according to their value of life on the abscissa and ordinate, the order in the vertical and horizontal direction is strictly monotonous (Figure 2, middle). Since there are 18 individual obstacles, the model has a total of 20 parameters, including the lane bias and starting lane coefficients.

**Figure 3:**
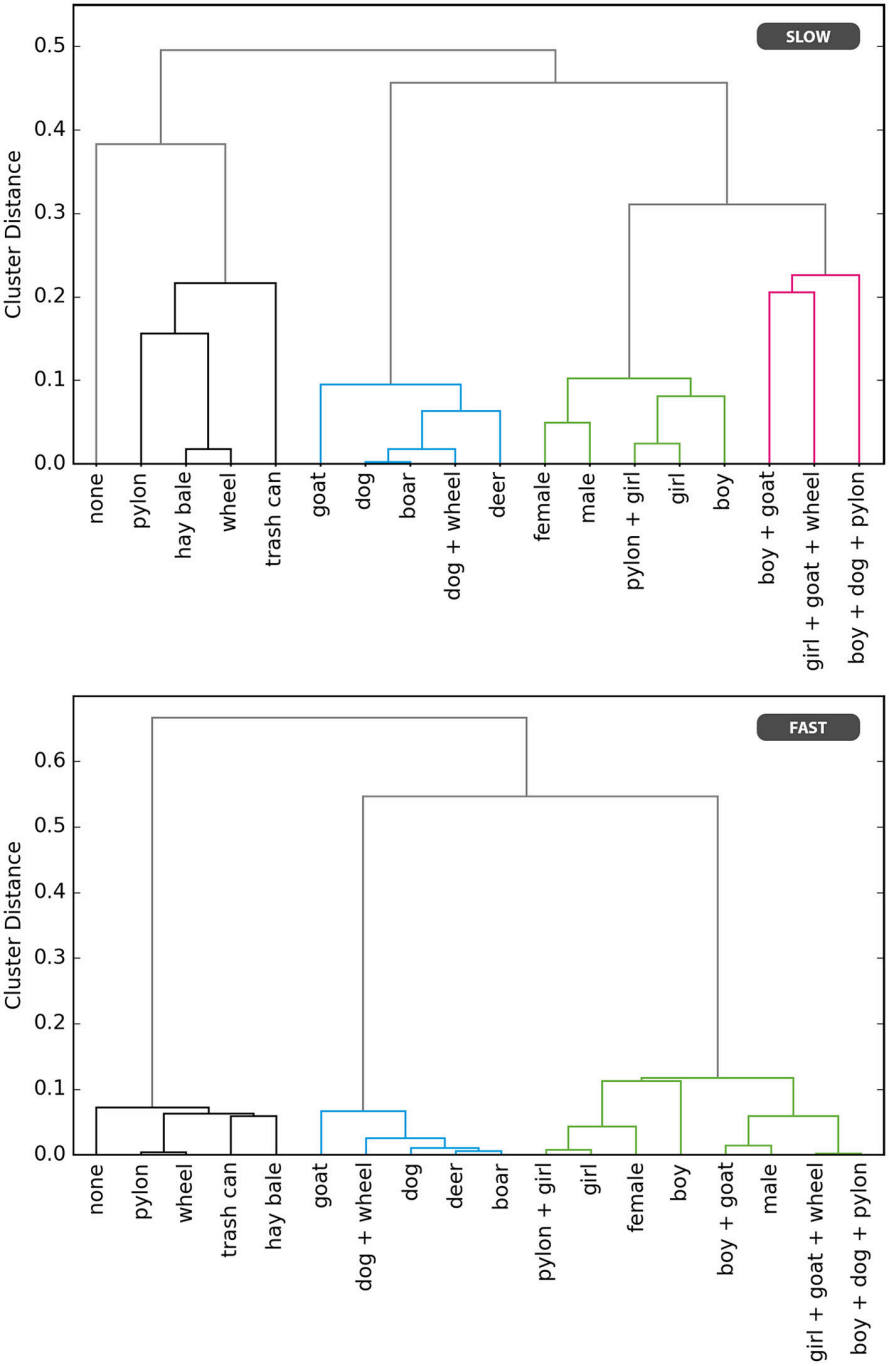
Dendrogram of bottom-up clustering, based on the observed frequencies with which each obstacle was spared (saved), for the slow and fast condition separately.

Similarly, in the cluster model, a trial is represented as *X*_*c*_ = ω_*rc*_ − ω_*lc*_ + *sω*_*s*_ + ω_*b*_, with ω_*rc*_ and ω_*lc*_ being the coefficients of the clusters that the obstacles are assigned to. We performed bottom-up clustering and subsequent model selection to derive the ideal number of clusters and cluster allocations of all presented obstacles for the cluster model (see Figure 3). Logistic regression models were first constructed and fitted for all possible numbers of clusters, ranging from 17 to 1. We then performed the model comparison via the Bayesian Information Criterion (BIC). In the slow condition, the five clusters model was found to be the best of the cluster models. Notably, its cluster allocations are perfectly in line with a categorization in none, inanimate objects, animals, humans, and groups of humans and animals. In the fast condition, a four cluster solution was found to be ideal, and its cluster allocations don't align perfectly with the aforementioned semantic categorization. This is likely the result of the higher error rate in the fast condition. In order to still allow for a comparison between both conditions, we chose to use the aforementioned semantic categorization in five clusters for the fast condition, as well. For both conditions, the cluster model thus has five parameters for the obstacle clusters, resulting in a total of only seven parameters, including the lane bias and starting lane coefficients. Figure 2 right shows its predictions in the slow condition. The model uses only one free parameter per cluster of obstacles, resulting in a block structure. Since all obstacles within a cluster are considered equal in value of life, the difference in the value of life is always exactly zero for within-cluster comparisons. Those decisions, therefore, depend entirely on the starting lane and overall lane preference.

All models were fitted using the logistic regression algorithm in the scikit-learn (version 0.17.1) toolbox for Python. We set the regularization strength to a very low value of 10^−9^ and based the model selection on prediction accuracy via 10-fold cross-validation, as well as the Bayesian Information Criterion.

#### Pairing model vs. obstacle model

In a first step, we compare the pairing and the obstacle models. When modeling the training data set, models with a (much) higher number of free parameters can describe the data better. However, in cross-validation, potential overfitting can lead to a reduced performance of the more detailed model. Indeed, with a prediction accuracy of 91.64% in the slow condition and 80.75% in the fast condition, the obstacle model is slightly superior to the detailed pairing model, with prediction accuracies of 89.33% and 78.77%, respectively. Despite our extensive data set with 909 trials per condition, the large number of parameters in the pairing model causes overfitting. This find translates to a much larger BIC value for the pairing model (see Table 1). Thus, our results strongly favor the obstacle model for its lower complexity and reduced risk of overfitting. This result, in combination with the high prediction accuracy of the obstacle model in the slow condition, confirms our first hypothesis, i.e., one-dimensional value-of-life-based models can adequately capture the ethical decisions we make in real life scenarios.

**Table 1:** BIC-values and prediction accuracy based on 10-fold cross-validation for the three models in the slow and fast condition.

**Model**	**SL**	**LB**	**Parameters**	**Slow**		**Fast**	
				**BIC**	**Accuracy**	**BIC**	**Accuracy**
Pairing	x	x	155	1349.122	0.8933	1563.629	0.7877
Obstacle	x	x	20	556.845	0.9164	770.827	0.8075
Cluster	x	x	7	497.198	0.9120	691.389	0.8053
Cluster		x	6	505.816	0.8922	685.797	0.8118
Cluster	x		6	**491.852**	0.9120	684.656	0.8053
Cluster			5	499.809	0.8636	**679.053**	0.8229

*SL, including the starting lane as predictor; LB, including a constant offset as predictor to model a lane bias. Bold values indicate the best model in the list*.

#### Obstacle model vs. cluster model

In the slow condition, the obstacle model's rankings of coefficient values within the categories mostly make sense, intuitively. For example, children are assigned higher values than adults (boy: 2.76, male adult: 2.12, corresponding to a 65.5% chance of saving the boy in a direct comparison with a male adult). Further, the dog is consistently found to be the most valuable of the animals. The prediction accuracies, however, are essentially even between the obstacle model (91.64%) and the cluster model (91.20%), with the cluster model scoring the lower BIC value, due to the reduced number of parameters (see Table 1). These findings are repeated in the fast condition. Prediction accuracies of the obstacle and cluster models are very close to each other (80.75 and 80.53%), and in terms of BIC values the cluster model is superior. We thus have to reject our second hypothesis, because the cluster model with five clusters is selected as superior to the obstacle model. In other words, we found no particular advantage of using obstacle-based predictors instead of category-based predictors.

#### Biases

To assess the two bias predictors' importance for the model, we ran another model comparison for three additional versions of the cluster model. All three additional versions were based on the above model, but the first variant dropped the starting lane predictor, the second variant dropped the lane bias, and the third variant dropped both predictors. In the slow condition, the cluster model omitting the lane bias, but including the starting lane predictor, scored the lowest BIC value of all models. Its prediction accuracy is the same as that of the previously assessed cluster model with both additional predictors (91.20%), making it the best explanatory model for the observations made in the slow condition (see Table 1). This is also reflected in the respective coefficients. When including the predictor of the lane bias, it was fit to a value of 0.15. The low value indicates only a very weak tendency to the left lane, which makes no significant contribution to the model fit. Thus, even in this rather realistic scenario, participants treated both lanes as equally valid driving lanes. The starting lane predictor was fitted to -0.47, indicating a reluctance to switch lanes in the face of a decision, constituting an omission bias. We can roughly quantify the extent of this reluctancy as being rather small, since coefficient differences between categories are in all cases magnitudes higher. The specific starting lane in a trial would therefore not affect the decisions in between category comparisons. It does, however, play a role in within-category decisions, as evidenced by the 4.8% gap in overall prediction accuracy between the cluster models with and without the starting lane as a predictor. In the fast condition, the best model, both in terms of prediction accuracy as well as BIC score, is the one omitting both bias predictors (see Table 1). By omitting the bias predictors, the prediction accuracy increases from 80.53 to 82.29%, exposing an overfit in the more complex model. In conclusion, the analysis of the bias predictors found lane preference to have no substantial influence on the decisions made in this paradigm, but did reveal an omission bias when facing similarly valued obstacles in the slow condition.

### Influence of increased time pressure

We will now turn to a direct comparison of the slow and fast condition, to evaluate the effects of increased time pressure. The most notable difference between the two conditions is the (estimated) error rate of 12.0% in the fast condition, marking a four-fold increase from the slow condition. As for the cause of the errors, we would expect an increased omission bias if the errors were caused by a mere failure to react in time. Interestingly, this was not the case. Instead, we found an omission bias only in the slow and not in the fast condition, indicating that errors in the fast condition were equally a result of staying in and switching into the lane of the more valuable obstacle. A major increase in error rate also substantially decreases the expected prediction accuracy even for a perfect model. This is reflected in the prediction accuracies for models in the fast condition, which are on average roughly 10% below those for the corresponding models in the slow condition.

**Figure 4:**
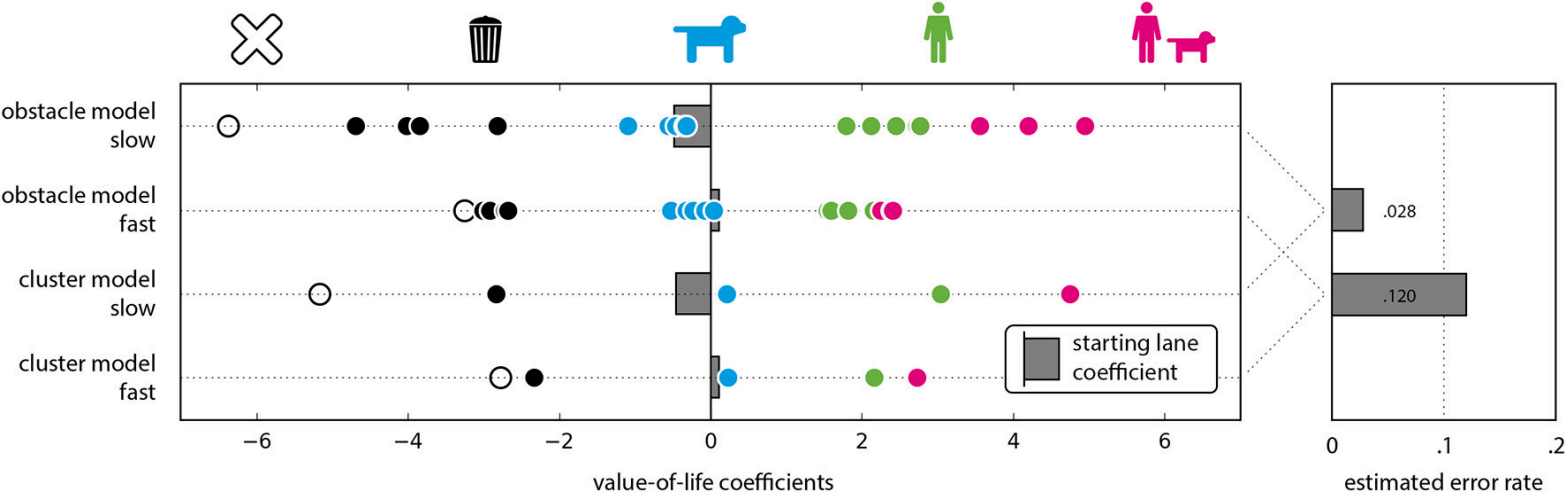
**(Left)** Value-of-life coefficients by condition. Pictograms and colors indicate the categories empty lane, inanimate objects, animals, humans, and groups of humans and animals (left to right). Starting lane coefficients depicted as gray bars. **(Right)** Relative frequency of “saving” the empty lane object, used as error rate estimates, for fast and slow condition separately.

In the cluster analysis, we found a four cluster model to yield the lowest BIC values in the fast condition, instead of the five cluster model found to be ideal in the slow condition. Moreover, the cluster assignments for some of the obstacles are also different, and do no longer match the semantic categories perfectly (see Figure 2). These findings are consistent with the influence of increased noise in the data, and can therefore also be ascribed to the increased error rates. Since there is no matching cluster model for both the slow and fast condition, we included a comparison of the cluster models based on the semantically defined categorizations in Figure 4, but decided to focus on the obstacle model in the remainder of this comparison. In the obstacle model, the coefficient range in the fast condition was reduced to 50% of that in the slow condition (see Figure 4). Specifically, the obstacles on the extreme sides of the spectrum—the empty lane and the groups of humans and animals—aren't separated well from the adjacent obstacle categories. To statistically confirm this observed difference, we used a nested model approach with log-likelihood ratio tests. For the nested model, we fitted the joint dataset of fast and slow conditions to the obstacle model using 19 predictors, i.e., the 18 obstacles plus the starting lane. For the larger nesting model, we added a second set of 19 predictors. These 19 were duplicates of the first 19 predictors, but were fitted only on the slow condition trials. Together, these two sets formed a model with 38 predictors in total. The log-likelihood ratio test between the nesting and nested model was significant (*p* = 0.037), showing that the reduction in parameters between the two significantly reduces model accuracy. In other words, the difference between the two conditions is large enough to justify the use of two completely separate sets of parameters to describe them. This confirms our third hypothesis, i.e., increased time pressure significantly decreases the consistency in the answering patterns.

Another notable difference between the two conditions is that we no longer observe a bias toward sacrificing the male adults in direct stand-offs with female adults. Instead, participants saved males in 4 out of 7 cases in the fast condition. The previously speculated tendency toward social desirability would likely rely on slower cognitive processes, and thus not come into effect in fast-paced intuitive decisions.

## Discussion

We investigated the capability of logistic regression-based value-of-life models to predict human ethical decisions in road traffic scenarios with forced choice decisions, juxtaposing a variety of possible obstacles, including humans, animals, and inanimate objects. The analysis incorporated various contextual and psychological factors influencing our moral decision-making in these situations, and examined in particular the effects of severe time pressure.

Our first hypothesis was that a one-dimensional value-of-life-based model fully captures the complexities of pairwise comparisons. With prediction accuracies well above 90% in the slow condition, and clearly outperforming the more complex pairing model, the obstacle model proved to be capable of accurately predicting the moral decisions made in the pairwise comparisons. The first hypothesis was thus confirmed. Note that since we used a wide range of obstacles, we cannot preclude some more complex effects happening on a more detailed level. One possible example of such an effect is the following: In the slow condition, the obstacle model shows male and female adults to have comparable value-of-life coefficients with a slight advantage for the males (2.12 vs. 1.79), predicting a 41.8% chance of sacrificing the male adult in a direct comparison. This prediction is based on all the trials it has seen, i.e., the full dataset including all possible combinations of the 18 obstacles. Still, adult males were actually sacrificed in 4 out of the 5 cases (80%) of direct comparisons between male and female adults. This observation is in line with Skulmowski et al. (2014), who also found males to be sacrificed more often in a direct comparison. Interestingly, the authors found the tendency to sacrifice males to be correlated with a general tendency to answer according to social desirability. In our study, the tendency to sacrifice males only pertains to the slow and not to the fast condition, which makes sense, if we assume that the effect is rooted in a tendency toward social desirability. Considerations of social desirability could be construed as part of the outcome-based system in Cushman's account of the dual-process theory, which is thought to be the slower one of the two processes. However, the low number of direct comparisons this figure is based on, and the exploratory nature of this find, dictate caution with respect to its interpretation. We consider it a leverage point for future research, but not a major result of this study.

Our second hypothesis was that within-category distinctions, for example between humans of different age, are an important factor in the decisions. This hypothesis could not be confirmed in this study, as the obstacle model failed to show an advantage over the cluster model in describing the collected data. However, there are hints at a meaningful structure within the clusters. For example, the obstacle model found children to have higher values than adults, and the dog, as the only common pet among the animals shown, to have the highest value within the animal cluster. Thus, given a larger data basis, we would still expect within-category distinctions to improve the predictions made by value-of-life models. In particular, we would expect age to play a role in human vs. human comparisons. Surveys by Cropper et al. (1994) and Johansson-Stenman and Martinsson (2008) have previously shown that the value we assign to someone's life decreases considerably with the person's age. To what degree these judgment-based findings would also be reflected in assessments of behavior is unclear, since judgment and behavior can yield dramatically different outcomes (Patil et al., 2014; Francis et al., 2016). Based on our findings, we speculate that the difference in value-of-life between people of different ages may be less pronounced in behavioral assessments, but more data is needed to clarify this point.

Irrespective of the exact outcome of such assessments, systems discriminating based on age, gender or other factors may be considered unacceptable by the public, as well as by lawmakers. Nevertheless, the idea of weighing lives against one another isn't generally rejected. As Bonnefon et al. (2016) showed, a majority of people would prefer a self-driving car acting in a utilitarian manner, at least when it isn't themselves, who are being sacrificed for the greater good. Independent of whether or not human lives should be weighed against one another, assigning different values of life to animals even seems to be the logical choice, judging from how differently we treat different species of animals in other aspects of life. Value-of-life models based on species would allow us to differentiate between common pets and other animals, and would give us a tool to deal with situations in which the death of an animal could be avoided by taking a minor risk of harm to a human.

Our third hypothesis was that ethical decisions would be less consistent across and within subjects when the time to react is reduced. This hypothesis was confirmed. The error rate was drastically increased, the cluster analysis revealed fewer clusters with slightly different cluster assignments, and the range of value-of-life coefficients was significantly reduced. However, we cannot deduct from our data whether the decisions made under time-pressure are in fact less clear-cut than decisions formed with more time for deliberation, or if the effect can be fully explained by the increased error rate. Still, a full second of time to react is a lot more than we typically encounter in real-life scenarios of this kind, and the weak consistency in the decision patterns is a sign that we are ill-equipped to make moral decisions quickly, even when the situation comes expectedly. We therefore argue that, under high time pressure, algorithmic decisions can be largely preferable to those made by humans.

Another noteworthy difference between the fast and slow condition concerns the omission bias, which we only found in the slow, but not in the fast condition. Participants were thus less likely to switch lanes and interfere in the situation when given more time to decide. This fact can be interpreted as a sign of a more deontological reasoning—choosing not to interfere in the situation, and possibly trying to reduce one's own guilt despite causing greater damage as a result. A tendency toward deontological reasoning with more time, however, conflicts with both Greene's and Cushman's accounts of the dual-process theory, as well as, e.g., Suter and Hertwig (2011), who found that more time to decide will cause a shift toward utilitarian responses. One possibly decisive difference between the present study, and most other studies touching on the aspect of time in moral decision-making, is the type of scenario used and the corresponding absolute response times. Typically, the scenarios used are relatively complex moral dilemmas, and response times lie in the 8–10 s range for short, and up to several minutes in the longer or unconstrained conditions (Greene et al., 2008; Suter and Hertwig, 2011; Paxton et al., 2012). In contrast, the reaction time windows of 4 and 1 s used in the present study rather represent a distinction between short deliberation and pure intuition. The fast condition may thus fall out of the dual-process theories scope.

In this study, we purposefully constructed a simple scenario with clearly defined outcomes, featuring the variables necessary to fit value-of-life models. With the general applicability of these value-of-life models established, a number of ensuing questions arise. For example, what influence a person's emotional and cognitive features have on their decision, how different probabilities of a collision or different expected crash severities affect our judgment, and how groups of multiple people or animals should be treated in such models. Moreover, the option of self-sacrifice has been prominently discussed in literature (Lin, 2014; Greene, 2016; Gogoll and Müller, 2017; Spitzer, 2016), and was assessed via questionnaire in Bonnefon et al. (2016), but hasn't been included in behavioral studies so far. We speculate that immersion and perceived presence may have a particularly strong influence on decisions that touch upon one's own well-being. Beyond this, considerations of fairness need to be addressed as well—for example, if one person is standing on a sidewalk and another has carelessly stepped onto the street. While the choice of a wide range of obstacles has proven helpful in understanding the big picture, more research is needed to answer open questions about effects happening within the categories. The design choices we made allowed us to focus on the applicability of value-of-life models, but the present study does not provide a fleshed-out model for implementation in self-driving cars. Instead, it constitutes a starting point from which to investigate systematically, how a variety of other factors may influence our moral decisions in this type of scenario and how they could be implemented.

A limiting factor for this study is the use of only one instance of each of the presented obstacles. We tried to select and create 3D models that are as prototypical as possible for their respective classes, but we cannot rule out that the specific appearance of the obstacles may have had an impact on the decisions, and by extension, the coefficient values assigned to the obstacles. Future studies or assessments that put more emphasis on the interpretation of single value-of-life coefficients, should include a variety of instances of each obstacle. Furthermore, larger and explicitly balanced samples would be needed to obtain models sufficiently representative of a society's moral judgment. Another fair point of criticism concerns the plausibility of the presented scenario. There was no option of braking during up to 4 s of decision time, and the car was keyboard-controlled and could only perform full lane switches. While there were good reasons for these design choices, namely to allow for enough decision time and to enforce a clear decision based on an unambiguous scenario, they limit the virtual world's authenticity and may hinder the subjects' immersion. Unfortunately, this issue seems unavoidable in controlled experimental settings. We believe that the virtual world implemented for this study nevertheless fulfills a high standard of authenticity overall, and, under the given constraints, illustrates the scenarios in question as close to reality as currently possible.

Future studies should further investigate the role of the presentation mode in this specific context. We argue that based on moral dilemma studies, a distinction between judgment and behavior may be justified. However, it remains to be seen if there is a seizable difference for specifically the kind of situations used in this study that justifies the special effort that goes into the design of a virtual reality environment. Finally, based on our findings, the influence of time pressure could be assessed in greater detail, expanding the considered time frames beyond the 1-4 s range.

## Conclusion

We argue that the high contextual dependency of moral decisions and the large number of ethically relevant decisions that self-driving cars will have to make, call for ethical models based on human decisions made in comparable situations. We showed that in the confined scope of unavoidable collisions in road traffic, simple value-of-life models approximate human moral decisions well. We thus argue that these models are a viable solution for real world applications in self-driving cars. With respect to trust in the public eye, their simplicity could constitute a key advantage over more sophisticated models, such as neural networks. Furthermore, regression models can include additional factors such as probabilities of injuries for the parties involved, and help to make reasonable decisions in situations where these differ greatly. They also provide an easy option to deal with a vast number of possible obstacles, by testing a few and making reasonable interpolations, e.g., for people of different age, taking away the requirement of assessing each conceivable obstacle individually. That being said, the modeling of within-cluster differences, e.g., between humans of different ages or between different species of animals, failed to improve upon the rather coarse cluster models. We further found time pressure, as an inherent feature to naturalistic portraits of the scenario in question, to considerably decrease the consistency in the decision patterns and call for more investigation of the effect of time pressure on moral decision-making. Overall, we argue that this line of research, despite being met with some skepticism (Johansson and Nilsson, 2016), is important to manufacturers and lawmakers. The sheer expected number of incidents where moral judgment comes into play creates a necessity for ethical decision-making systems in self-driving cars (Goodall, 2014a). We therefore hope to see more efforts toward establishing a sound basis for the methodology of empirically assessing human ethics in the future, as the topic is becoming increasingly important with more advances in technology.

## Author contributions

LS: Leading role in planning, implementation, data acquisition and data analysis. Writer of the paper. RG: Involved in planning, data acquisition and data analysis. Gave feedback to earlier versions of the paper. GP and PK: Involved in planning, data acquisition and data analysis, and gave feedback to earlier versions of the paper.

### Conflict of interest statement

The authors declare that the research was conducted in the absence of any commercial or financial relationships that could be construed as a potential conflict of interest.
